# A Wavelength Modulation Spectroscopy-Based Methane Flux Sensor for Quantification of Venting Sources at Oil and Gas Sites

**DOI:** 10.3390/s22114175

**Published:** 2022-05-31

**Authors:** Simon A. Festa-Bianchet, Scott P. Seymour, David R. Tyner, Matthew R. Johnson

**Affiliations:** Energy & Emissions Research Laboratory, Department of Mechanical and Aerospace Engineering, Carleton University, Ottawa, ON K1S 5B6, Canada; simonfestabianchet@cmail.carleton.ca (S.A.F.-B.); scottseymour@cmail.carleton.ca (S.P.S.); drtyner@gmail.com (D.R.T.)

**Keywords:** methane, emission spectroscopy, mass flow, venting, oil and gas sector, hazardous locations, storage tanks, Doppler shift

## Abstract

An optical sensor employing tunable diode laser absorption spectroscopy with wavelength modulation and 2*f* harmonic detection was designed, prototyped, and tested for applications in quantifying methane emissions from vent sources in the oil and gas sector. The methane absorption line at 6026.23 cm^–1^ (1659.41 nm) was used to measure both flow velocity and methane volume fraction, enabling direct measurement of the methane emission rate. Two configurations of the sensor were designed, tested, and compared; the first used a fully fiber-coupled cell with multimode fibers to re-collimate the laser beams, while the second used directly irradiated photodetectors protected by Zener barriers. Importantly, both configurations were designed to enable measurements within regulated Class I / Zone 0 hazardous locations, in which explosive gases are expected during normal operations. Controlled flows with methane volume fractions of 0 to 100% and a velocity range of 0 to 4 m/s were used to characterize sensor performance at a 1 Hz sampling rate. The measurement error in the methane volume fraction was less than 10,000 ppm (1%) across the studied range for both configurations. The short-term velocity measurement error with pure methane was <0.3 m/s with a standard deviation of 0.14 m/s for the fiber-coupled configuration and <0.15 m/s with a standard deviation of 0.07 m/s for the directly irradiated detector configuration. However, modal noise in the multimode fibers of the first configuration contributed to an unstable performance that was highly sensitive to mechanical disturbances. The second configuration showed good potential for an industrial sensor, successfully quantifying methane flow rates up to 11 kg/h within ±2.1 kg/h at 95% confidence over a range of methane fractions from 25–100%, and as low as ±0.85 kg/h in scenarios where the source methane fraction is initially unknown within this range and otherwise invariant.

## 1. Introduction

Methane emissions at upstream oil and gas production sites, in particular from atmospheric vents on storage tanks and well casings, have been identified as a potentially underestimated contributor to total industry emissions [1,2,3,4,5]. Vented methane volumes from these sources are challenging to measure directly, as the flows may be both intermittent and transient and the composition (methane fraction) in the emitted gas may vary in time depending on the processes driving the emissions. Moreover, the nature of these sources and the facilities within which they operate mean that measurements must be made in Class I / Zone 0/1 hazardous locations, where explosive gases may be present as part of routine operations. For these reasons, vented methane emissions at oil wells are typically estimated using a “gas–oil ratio” (GOR), which relates the estimated volumes of produced gas to measured oil volumes at a well and an assumed or infrequently measured gas composition. This is problematic for a number of reasons, as transient emissions are likely to be missed, the GOR itself is difficult to measure accurately, and at heavy-oil sites or sites with associated gas (i.e., gas that exists as a separate phase within the deposit) the GOR may vary widely due to changes in the underground deposit as oil is removed [6]. 

An optical methane flux sensor based on measuring the Doppler shift using tunable diode laser absorption spectroscopy with wavelength modulation is presented here as a potential solution to this measurement problem. First proposed by Measures [7] as a technique for monitoring supersonic flows, optical mass flux sensors have been demonstrated in shock tube experiments with velocities ranging from 500 to 1500 m/s. Probed species have included NO [8], O_2_ [9], and H_2_O [10]. Other studies [11,12,13] have investigated scramjet engine performance by measuring water vapor at supersonic velocities, and a mass flux sensor for hypersonic flight using ambient O_2_ has been developed as well [14,15,16].

However, applications of this approach at subsonic gas velocities have been limited. Lyle et al. [17,18] investigated aero-engine inlet characteristics using a hybrid technique which combined direct absorption to measure the density, pressure, and temperature of oxygen and wavelength modulation spectroscopy to measure flow velocity. This work was expanded on by Chang et al. [19], who developed a mass flux sensor targeting the water vapor produced by combustion facilities. Validation results were presented for a velocity range of 2.5 to 18 m/s in a wind tunnel with ambient humidity, with a reported precision error of less than 0.5 m/s. Kurtz et al. [20] developed an aircraft flight speed sensor using O_2_ that was capable of operating at an altitude of up to 12 km.

Notably, in each of these studies, the volume fractions of the target species were either fixed or constrained within a narrow range of absorbances. In contrast, for our intended application of measuring key oil and gas sector point sources, methane volume fractions are expected to span at least 50–99% and potentially vary over time. The velocities of interest are lower than in previous studies, with a target range of 0.2 to >5.7 m/s in a nominal 2-inch diameter cross-section corresponding to methane mass flows of ~1 to >32 kg/h [4]. To the authors’ best knowledge, the present work is the first to use an optical mass flux sensor to quantify methane flows. Finally, the described system enables deployment in Class 1 / Zone 0 hazardous locations with an intrinsically safe optical measurement head. This safety feature is essential for the target application of directly measuring methane vent sources in the upstream oil and gas sector. 

## 2. Materials and Methods

Wavelength modulation spectroscopy is used to measure both CH_4_ mole fraction and bulk flow velocity, while absolute pressure and temperature of the gas are measured using intrinsically safe auxiliary sensors. The CH_4_ mole fraction, pressure, and temperature are combined to calculate CH_4_ density. The product of velocity and density yields the methane mass flux.

### 2.1. Wavelength Modulation Spectroscopy

Tunable diode laser absorption spectroscopy (TDLAS) targets distinct spectral absorption band(s) of a target gas species to measure various properties of the atoms or molecules causing the absorption. Tunable diode lasers are used as a source of narrow bandwidth light, and photodetectors are used to measure changes in light intensity after interacting with the fluid containing the target molecule. For atmospheric gases near STP, scattering effects can generally be ignored [21] and the Beer–Lambert–Bouguer law can be applied to relate the transmittance, τ(v), of light at frequency v [cm^–1^] (i.e., the ratio of the optical intensity after absorption, *I* [W], and the unattenuated intensity, $I_{0}$ [W]), to the number density of the absorbing particles, $N_{i}$ [particles/m^3^], the optical path length, L [m], and the absorption cross-section, σ [m^2^/molecule]:

(1) $$\tau \left(v\right)=\left(\frac{I}{I_{0}}\right)=\exp\left(-N_{i}L\sigma \left(p,T,q,v\right)\right)=\exp\left(-\frac{qpL}{kT}\sigma \left(p,T,q,v\right)\right)$$

where p is pressure [Pa], T is temperature [K], and $q=N_{i}/N_{tot}$ is the number fraction (“volume mixing ratio”) of the absorbing species, i (in this case, methane). This is further simplified by substituting the ideal gas law in the form of $N_{tot}=\frac{p}{kT}=\frac{N_{i}}{q}$, where k is the Boltzmann constant (1.3806 × 10^–23^ [kg·m^2^]/[s^2^·K]).

The tunable diode laser in this sensor (Eblana, EP1662-3-DM-B06-FA) is tuned to the R 1 F1(1) absorption line of CH_4_ with center frequency 6026.23 cm^–1^ (1659.41 nm) and a stretching vibration mode in the 2$v_{3}$(F2) band of the Tetradecad region from 4760 to 6250 cm^–1^ [22]. The 2$v_{3}$ region of methane has been used in many remote sensing applications [23,24,25,26]. As further described below, the relatively short optical path length of 10.5 cm, transecting the 5.1 cm (2”) diameter measurement cell, guided the selection of a strong yet isolated CH_4_ absorption line free from potential interference by common atmospheric gases and other species found in associated gas (natural gas) at upstream production sites. This sensor makes use of Wavelength Modulation Spectroscopy (WMS), where the laser’s current, and thus its wavelength, is varied by a high-frequency sinusoidal modulation. This wavelength modulation is superimposed on a slower triangle waveform to scan over the linewidth of the absorption line. The theory of WMS has been well-described in the literature [27,28,29,30,31,32,33]. In this work, phase-insensitive detection is used to extract the total second harmonic (2*f*) feature of the absorption line. This is achieved by calculating the root-sum square of the in-phase and quadrature components of the second harmonic.

A pre-computed lookup table (LUT) of the relevant Fourier series coefficient was calculated using a Lorentzian lineshape, with $\bar{v}$ fixed on the theoretical line center wavelength of 6026.23 cm^–1^, path length L of 10.5 cm, and spectroscopic data from the HITRAN database [34]. This four-dimensional LUT covers a temperature range of –30 to +70 °C in steps of 1 °C, a pressure range of 90 to 105 kPa in steps of 2.5 kPa, and CH_4_ volume fractions from 0 to 100% in steps of 1%. To account for potential changes in return intensity at each detector, e.g., due to thermally-induced misalignment or bulk attenuation from entrained aerosols or fouling of the windows, the LUT must be scaled by the absorption-free intensity ($I_{0}$) for accurate results. This absorption-free intensity was estimated using the 0f harmonic, which is calculated similarly to the 2f harmonic except that the detected signals are multiplied by 1 (i.e., cos(0)), thereby preserving only the DC components after the low-pass filter is applied. In an optically thin scenario (absorption < 5%), the 0f can be directly used as a substitute for $I_{0}$ with minimal error [35]. In the present case, absorption is as high as 45%, and the drop in intensity in the 0f signal due to the presence of methane is thus mitigated by linearly interpolating between the highest and lowest values of the 0f signal, removing the portion of the signal most affected by absorption. The mean value of this interpolated signal was then used as an estimate of $I_{0}$. The theoretical amplitude of the 2f signal can then be evaluated for a known temperature and pressure and compared to the experimentally observed amplitude. Finally, a second-order polynomial calibration is applied to the theoretically evaluated volume fraction from each detector, and the mean value of these two results is reported as the measured methane volume fraction. This empirical calibration is included to account for errors in estimating $I_{0}$, as described above.

Other WMS processing techniques which make use of multiple harmonics, including 1*f*-normalized harmonics, have been described for volume fraction measurements of various gases [36,37]. However, 1f-normalization is not well-suited for optically thick conditions such as the present case, as the 1f becomes distorted by the strong optical absorption, leading to proportional errors in the 2*f*/1*f* signal (e.g., an absorption of 45% would yield an induced error of ~45% to the measured volume fraction) [38]. Thus, we adopted the 2*f*/0*f* approach. Moreover, as described in the following sections, the velocity measurement rather than the volume fraction measurement proved to be the limiting factor in sensor accuracy.

### 2.2. Velocity Methodology

The bulk velocity of a fluid can be measured by detecting the Doppler shift in the absorption frequency of a tuned laser beam with a component parallel to the flow direction [7]. The magnitude of this frequency shift, Δv [cm^–1^], is provided by

(2) $$\Delta v=\frac{v_{0}U}{c}\cos\left(\theta \right)$$

where $v_{0}$ is the unshifted line center frequency [cm^–1^], U is the fluid velocity [m/s], c is the speed of light (3 × 10^8^ [m/s]), and θ [deg] is the angle between the laser beam and the fluid velocity direction vector. Measurement accuracy can be improved by using a cross beam arrangement with two beams directed in opposite directions, effectively doubling the Doppler shift magnitude for a given flow velocity and intersection angle. This configuration eliminates the requirement for an absolute laser wavelength reference, as the position of the original unshifted absorption peak is not required. The fluid velocity is then calculated from the difference in the relative position of the 2*f* peaks of the two beams, $\Delta v_{1-2}$ [cm^–1^]:

(3) $$U=\frac{\Delta v_{1-2}c}{2v_{0}\cos\left(\theta \right)}$$

This difference in 2*f* peak positions is determined using a cross-correlation algorithm applied to synchronous pairs of 2*f* signals. Other methods of determining the Doppler shift were investigated, including various peak fitting algorithms such as a quadratic or spline fit. However, the cross-correlation algorithm showed the most reliable and lowest noise performance across the range of absorption levels investigated. Due to background signals not related to the absorption of methane (e.g., interference fringes) the measured velocity will typically have an error or offset that should be removed to improve precision [39]. The amplitude and temporal stability of these background signals will dictate how often this correction must be made, or in the worst case, whether the measurement is feasible for the intended application. While removal of background signals requires an absorption-free measurement, the velocity offset is comparatively easier to acquire in a realistic measurement setting by momentarily diverting flow away from the measurement tube. Finally, a linear calibration factor can be applied to the measured velocity to compensate for the intersection of the laser beams with a non-uniform flow profile across the measurement cell.

### 2.3. Sensor Description

**Figure 1:**
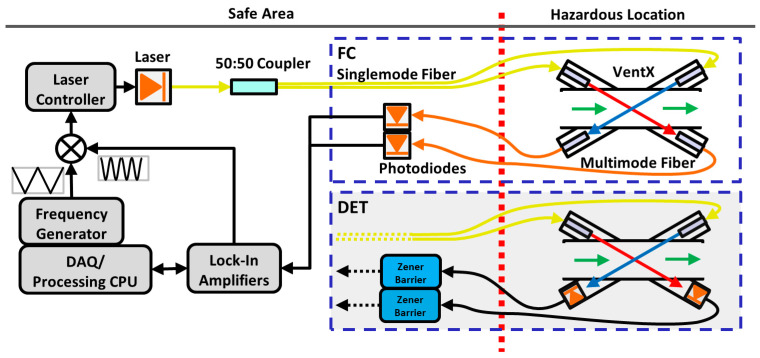
Schematic of the methane flux sensor, illustrating both the fully optical configuration (FC) and detector configuration (DET). A dotted red line separates the Safe Area on the left from the Hazardous Location on the right. Auxiliary intrinsically safe temperature and pressure transducers (not pictured) are included with the flow cell. The two light beams are collimated and arranged in a cross-beam pattern such that one beam is Doppler-shifted to a higher frequency and the other to a lower frequency when gas is flowing through the cell (green arrows). Signals return to the Safe Area as either optical signals in multimode fibers (FC) or electrical signals in conventional cables (DET).

Figure 1 shows a schematic of the sensor, which is conceptually divided into two sub-assemblies: an electro-optical base station, and a flow cell/pipe (labelled as “VentX”) through which the measured gas flows. The former must be in a safe area (i.e., non-hazardous), while the latter can be installed in a Zone 0 hazardous location, such as the immediate vicinity of a methane vent. The base station was housed in a Pelican–Hardigg rack-mount case to allow for easy transportation and protection in the field. A PC (PXIe 8880, National Instruments, Austin, TX, USA) was used to operate the sensor as well as to process and store data. A pair of digital lock-in amplifiers (MFLI, Zurich Instruments, Zürich, Switzerland) modulated the laser diode, while a frequency generator (PXI 5406, National Instruments, Austin, TX, USA) provided the low frequency sweep signal. Individual sweeps were demodulated and averaged on the lock-in amplifiers before being transferred to the PC at 1 Hz. The modulation frequency was 10 kHz and the sweep frequency was 30 Hz. The combined sweep and modulation signals were fed into a laser temperature and current controller (LDC500, Stanford Research Systems, Sunnyvale, CA, USA), which directly modulated the laser’s current and thus its output wavelength. A fiber optic 1 × 2 coupler with a 50:50 split ratio (TW1550R5A1, Thorlabs, Newton, MA, USA) split the laser output intensity into two 30-m long SMF-28 ruggedized fiber optic cables (Custom, O-m6 Technologies, Mirabel, QC, Canada) connected to the flow cell. 

Two configurations of the flow cell were investigated, both of which make use of intrinsic safety concepts to allow for use in hazardous environments. In the “fiber-coupled” (FC) configuration (Figure 1 top right dash-outlined box), the optical signal was returned to the base station using 600-μm core step-index fiber in ruggedized cables 3 m in length with SMA connectors (Custom, Thorlabs, Newton, MA, USA). The optical signals were converted to electrical signals by amplified InGaAs photodetectors (PDA10CS, Thorlabs, Newton, MA, USA) located in the base station. The FC configuration is similar to sensors described in other works which made use of multimode fibers to efficiently capture and transmit the light back from the measurement area [13,39]. Interestingly, Miller et al. [39] removed their multimode fibers mid-way through their experimentation by placing their detectors directly in the beam path. This modification was made after observing what they described as beam-steering due to vibrations in the fully optical configuration, which caused significant measurement error. Single-mode fibers were initially considered for the recoupling leg; however, in early mock-ups of the flow cell a stable alignment was extremely hard to achieve and the cell proved extremely sensitive to minor disturbances, to the extent that a light bump could result in a complete loss of recoupling. Additionally, alignment could not be maintained between disassembly and reassembly of the cell, which is desirable for a field-deployable system. For these reasons, single-mode recoupling was not considered further despite its potential advantages with respect to signal quality over multimode fibers.

In the “detector configuration” (DET), photodiodes (SM1PD4A, Thorlabs, Newton, MA, USA) were placed in the flow cell (within the hazardous zone) and directly illuminated by the laser beams after transmission through the gas. The current signals from these photodiodes were routed via 30-m long coax cables through low-resistance Zener barriers (Z757, Pepperl & Fuchs, Mannheim, Germany), which maintained the intrinsic safety of the measurement cell. The output signals from the Zener barriers were then fed into the current inputs of the lock-in amplifiers. 

**Figure 2:**
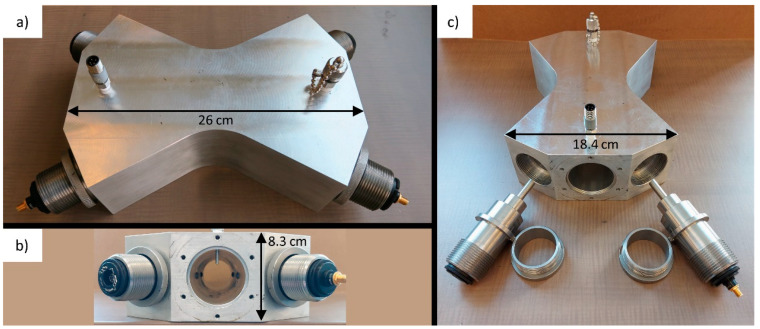
(**a**) Photo showing the assembled flow cell in the DET configuration. Fibers and electrical cables have been removed for clarity, as have the inserts’ rear caps with cable glands. (**b**) View of the central bore of the flow cell, showing the near unobstructed flow path. The RTD probe can be seen protruding into the flow path from above, along with the tips of the four inserts at the horizontal centerline. (**c**) In this photo, the inserts have been removed from the flow cell to show their form.

The flow cell (Figure 2) was machined from aluminum and features a 2.067” inner diameter bore, a common size for vent lines at oil and gas production sites. This flow-through cell was designed to add negligible back pressure to the system under test, a critical requirement when instrumenting fixed roof storage tanks. The cell holds four removable stainless steel inserts, which contained either collimators mounted in kinematic mounts or a rigidly mounted photodetector. These inserts terminated in cable glands sealed around the optical and/or electrical cables. O-rings between the inserts and the aluminum cell ensured a gas-tight seal, and the inserts were retained by hand-tightened knurled rings. 

In both configurations, the laser light was collimated into 1.29 mm diameter beams (1/e^2^) using singlet collimators (F110APC-1550, Thorlabs, Newton, MA, USA) fixed in kinematic mounts (KAD11F, Thorlabs, Newton, MA, USA), which were threaded into the inserts. The kinematic mounts helped to maximize return intensity, compensating for pointing error due to collimator variability and machining tolerances. The clear aperture through the flow cell was 3.76 mm, with custom fused-silica wedged windows glued into a socket at the tip of the inserts to provide optical access. These windows had a 30 arcmin wedge and were mounted at an angle of 15° to the beam path to reduce etalon effects. After interacting with the gas flowing through the cell, the laser light entered an identical insert through its wedged window. In the FC configuration, the light was re-coupled into multimode fibers using singlet collimators (F240SMA-1550, Thorlabs, Newton, MA, USA). In the DET configuration, the light directly irradiated the photodetectors. An intrinsically safe pressure transmitter (A4SAM0242E030#A, Ashcroft, Stratford, CT, USA) with 0.25% of span (30 psia) accuracy and ≤1% temperature effect and a four-wire Class A RTD (Pt100, Evolution Sensors, Mullica Hill, NJ, USA) were threaded into the cell to provide gas absolute pressure and temperature, respectively. The RTD temperature was converted to a 4–20 mA signal using an intrinsically safe temperature transmitter (T15, WIKA Instruments, Edmonton, AB, Canada) with an accuracy of 0.2 K and a temperature coefficient of ≤±0.1 K/10 K.

## 3. Results

The performance of the methane mass flux sensor in both configurations was characterized in a laboratory setting using controlled methane releases at various flow rates and compositions. Thermal mass flow controllers (EL-FLOW, Bronkhorst, Ruurlo, The Netherlands) were used to meter methane and nitrogen to produce methane mass flows of up to 20 kg/h at methane volume fractions varying from 0 to 100%. This corresponded to an absorbance range of 0 to 0.61 and bulk velocities of up to 4 m/s in the flow cell.

### 3.1. Methane Volume Fraction

**Figure 3:**
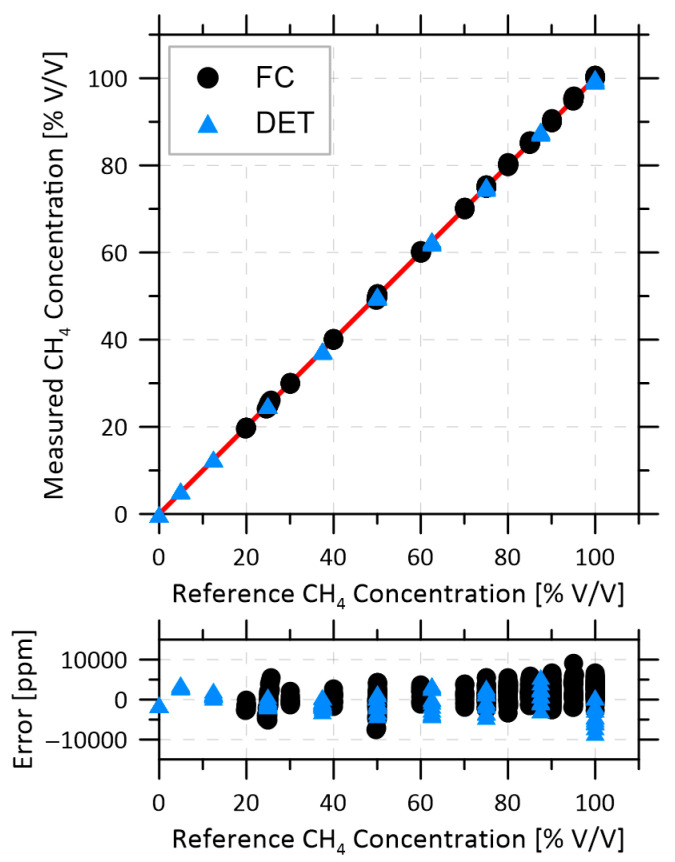
Volume fraction measurements for both configurations, with the ideal 1:1 line in red. Measurement error in ppm is shown at the bottom.

A sample of methane volume fraction measured by each configuration is plotted in Figure 3 against the methane volume fraction recorded by the mass flow controllers. The red line shows the desired 1:1 trend. Across all measurements, the volume fraction error was less than 10,000 ppm (1%) for both FC and DET configurations. This demonstrates the accuracy of the present 2*f*/0*f* approach using a 4D LUT with empirical correction, which effectively compensates for the non-linear relationship between volume fraction and 2*f* peak height previously reported for similarly large absorbance and optically thick conditions [36,40]. It is possible that the accuracy of the volume fraction measurement could be further improved with subtraction of the absorption-free background 2*f* signals or 1*f*- rather than 0*f*-normalization [41]. However, as previously discussed, the challenges in acquiring the absorption-free background in a field setting preclude the use of any background subtraction technique, and 1*f*-normalization may not perform with absorption fractions this high [38]. The presently achieved precision of 1% methane volume fraction is more than sufficient for the intended application of quantifying vented methane emissions in the oil and gas sector.

### 3.2. Flow Velocity

**Figure 4:**
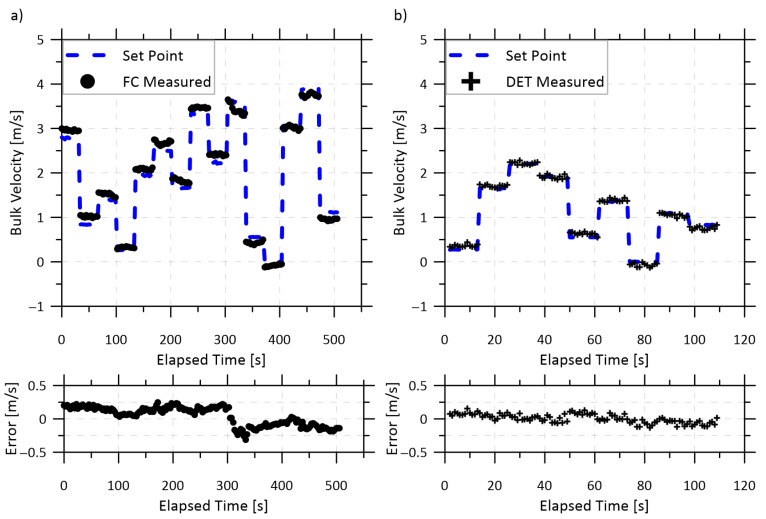
Time-resolved velocity of pure methane compared to set points as reported by mass flow controllers for (**a**) the FC configuration and (**b**) the DET configuration. The lower plots show the measurement error for their respective sequences.

**Figure 5:**
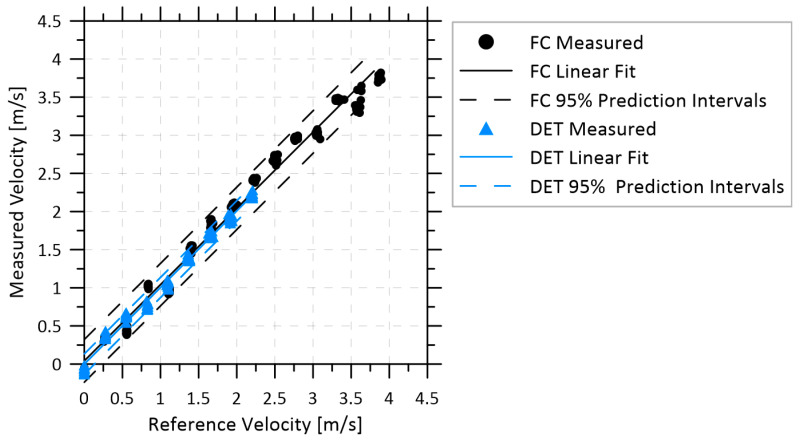
Velocity data from Figure 4 for both configurations, plotted as measured velocity from the sensor versus reference velocity from the mass flow controllers. Linear regressions of these data and prediction intervals at 95% confidence are shown for both configurations.

Figure 4 shows a typical velocity measurement using either the FC or DET configurations with pure methane at velocities up to 3.9 m/s (mass flow of 21.8 kg/h). These FC and DET data were corrected by the measured velocity offsets of 4.2 and 0.34 m/s, respectively, at zero flow shortly before the tests were started. The measurement errors are shown in the bottom graphs, which remained below 0.3 m/s in the FC configuration and below 0.15 m/s in the DET configuration. The sample standard deviation of the velocity error was 0.14 m/s in the FC configuration and 0.07 m/s in the DET configuration. These results compare favorably to those achieved by Lyle et al. [17] for measuring oxygen; they reported a measurement accuracy of 0.25 m/s and a standard deviation of 0.22 m/s in low-speed (0 to 23 m/s) wind-tunnel testing. The results from that study as well as from other sensors which have used WMS to measure subsonic flow velocities are compared in Table 1. A better measure of the accuracy of the velocity measurement is the prediction interval at 95% confidence, which is plotted in Figure 5 and shows that the FC and DET configurations can accurately quantify velocities measured at 1 Hz within ±0.32 and 0.16 m/s, respectively. However, the performance of the FC system was sensitive to disturbances of the multimode return fibers, as elaborated below, and for both systems the velocity measurement precision degraded with reduced methane fraction. 

**Figure 6:**
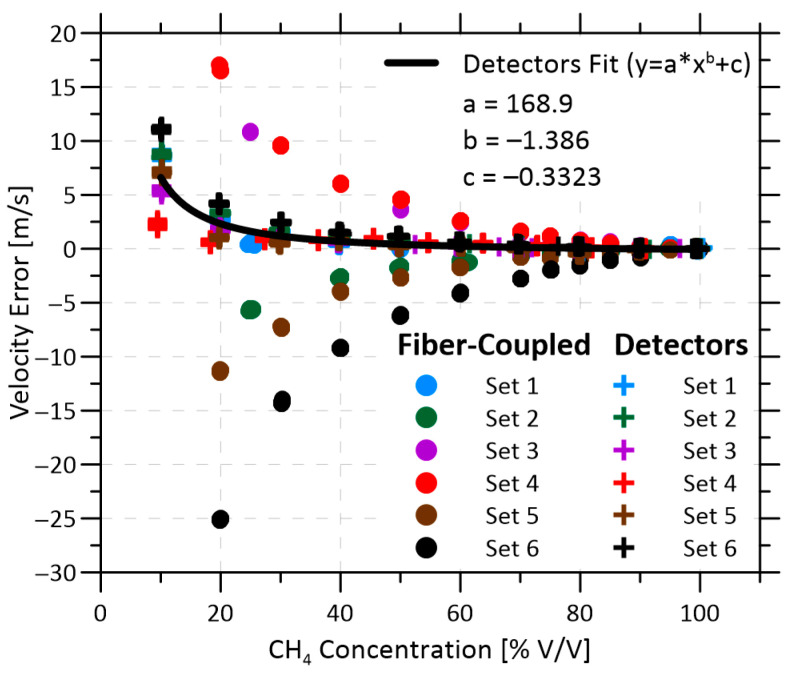
Velocity error as a function of methane volume fraction collected over six different tests for both the FC (dots) and DET (crosses) configurations. For the FC sets, manual disturbances were applied to the multimode fibers between each run. The DET sets were collected twice a day over three days. The velocities used ranged from 0 to 1.5 m/s, scrambled amongst all test volume fractions. Each set is zeroed by the average velocity at 100% methane at the beginning of that test.

Figure 6 compiles six independent datasets for each of the FC and DET configurations (dots and crosses, respectively) to show the absolute error in measured velocity as a function of the methane volume fraction. All data are presented as 1-s averages. For each of these runs, identified by separate colours, the measured zero-velocity offset bias at 100% methane has been subtracted to isolate the volume fraction-dependent bias error. Between each FC data set, the multimode fiber optic cable was manually disturbed and then allowed to rest for 30 min. Between each DET data set, the measurement cell was disassembled, the inserts removed, and the kinematic mounts for the collimators disturbed and re-aligned. Figure 6 shows that the volume fraction-dependent bias error can be significant. To the authors’ knowledge, this type of bias error has not been considered in previous studies, which have predominantly reported measurements at relatively constant absorbances. 

For the FC configuration, there is significant variability among the different runs, and this increases as the methane fraction is reduced. However, within each run, this offset is notably stable and independent of the measured velocity. As can be seen on close inspection of Figure 6, the “points” within each run are in fact a collection of independently plotted bias errors measured at one or more prescribed velocities ranging from 0–1.5 m/s. The close overlap of these points for each run shows that the trends are independent of velocity and that there is a high degree of repeatability within each run. 

Although the volume fraction-dependent velocity offsets were stable and repeatable across the different runs of the DET configuration, the opposite was true for the FC configuration. For the FC configuration, the error was sensitive to the handling of the multimode return fibers and was not repeatable. The error in the velocity measurement (represented by the spread of results for the different runs) grows non-linearly as the methane volume fraction is reduced, spanning 10.5 m/s at 50% methane and 41.5 m/s at 25% methane. Curiously, the trends for each run are quite smooth, suggesting that the errors are not random and that there is rather a specific volume fraction-dependent bias for each run related to the handling of the fibers. As further discussed below, this behavior is attributed to wavelength-dependent background signals; these have a larger impact on the velocity as the absorbance, and thus the signal to noise ratio, drops. This behavior is problematic for measurement applications where the methane volume fraction varies, as can occur in measurements of vent flows from uncontrolled oil storage tanks.

**Figure 7:**
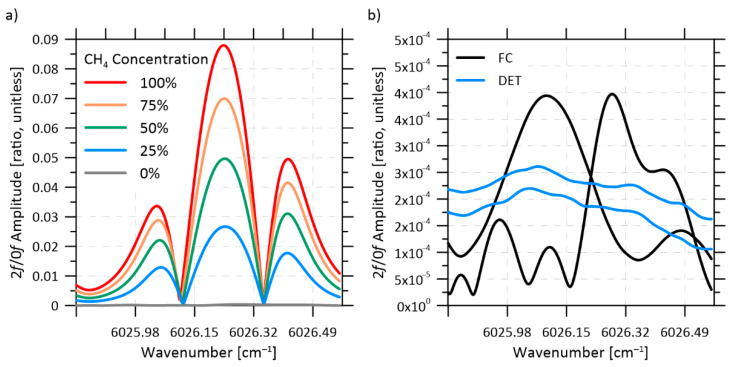
(**a**) 0*f* normalized 2*f* signals (2*f*/0*f*) over a range of methane volume fractions, using the FC configuration. (**b**) Background 2*f*/0*f* signals when the measurement cell was filled with nitrogen, for both the FC (black) and DET (blue) configurations. Both subplots only show the first half of the triangular sweep period.

Figure 7a shows the 2*f* harmonic normalized by the 0*f* signal for a range of methane volume fraction from 0–100% in the FC configuration. Figure 7b plots sample background 2*f*/0*f* signals at 0% CH_4_, when the measurement cell was purged with nitrogen. These background signals are less than 1% of the peak amplitude of the 2*f*/0*f* signal at 100% CH_4_. The origins of these background signals include non-absorption losses such as etalons caused by reflective surfaces and modal noise. While the flow cell was designed to minimize the former as much as possible, modal noise has been identified as a limitation of optical devices, such as spectrograms, which employ multimode fibers to recouple light and direct it to a photodetector [42,43]. Modal noise occurs when the multitude of modes in the multimode fiber interact at the fiber–air interface, creating a complex interference pattern which varies the light intensity reaching the photodetector. This output pattern is affected by wavelength, input conditions, temperature, and geometry of the fiber. Critically, this modal noise differs between the two return lines, as the light from the upstream and downstream beams travels within its respective multimode fiber. This difference is illustrated in Figure 7b, where the background signals in the FC configuration are noticeably different between the two detected signals. Manually re-positioning the multimode fibers irreversibly changes the interference pattern, and thus the velocity error. 

The amplitude of these background signals is sufficient to affect the velocity measurement, which is particularly sensitive to the shape of the processed signals. The detrimental effect of background signals can be mitigated if these unwanted signals can be recorded and then subtracted from measured spectra [44]. However, such a background subtraction technique is reliant on the stability of these background signals, and is unlikely to be practical for an in situ sensor intended to take long-term measurements. Additionally, a purging system to enable recording of absorption-free background signals is unlikely to be feasible in the field. A potential solution specific to modal noise is mechanical fiber excitation [42], where the multimode fibers are cyclically disturbed by a vibrating membrane or rotating arm with the goal of scrambling the modal noise to such an extent that its impact is averaged out. However, such a system would add undesirable complexity to the sensor and has never previously been applied to a velocity measurement. For these reasons, we concluded that a fully fiber-coupled measurement pipe is not suitable for our application, despite showing promise in static conditions. 

In contrast, results of Figure 6 show that the DET configuration is comparatively stable even with the necessary requirement of routing the unamplified photodiode signals through an isolation barrier to maintain the hazardous location rating of the sensor head. Additionally, Set 4 was collected after heating the entire flow cell to 33 °C, ~10 °C warmer than the other five sets, which were collected at lab temperatures around 23 °C. This increased stability observed in the DET configuration is primarily attributed to the elimination of the modal noise generated by multimode fibers. As shown in Figure 7b, the remaining background signals on each line of the DET configuration (blue curves) are lower in magnitude and more consistent than in the FC configuration. These residual background signals are likely caused by components common to both configurations, including: the splitter, the windows in the flow cell, launch collimators, and single mode fiber connections. This translates to much smaller velocity offsets, e.g., ≤1 m/s with the DET system vs. ≤6.2 m/s for the FC system at ≥50% methane volume fraction. 

### 3.3. Methane Mass Flow Rate Accuracy in Different Field Measurement Scenarios

**Figure 8:**
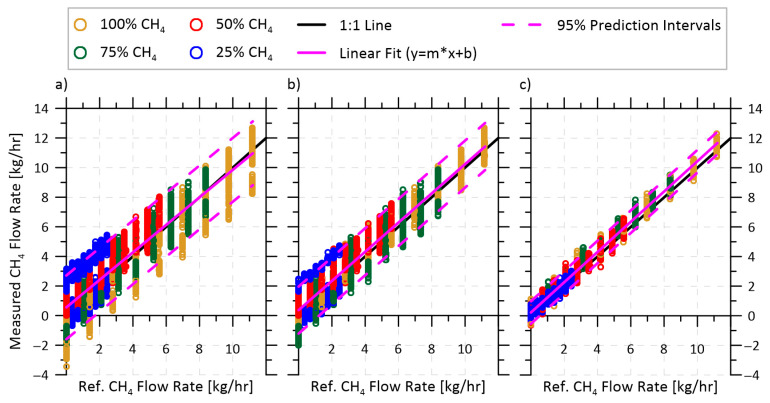
Achievable prediction intervals at 95% confidence when measuring methane mass flow rates in three scenarios: (**a**) extremely variable source methane volume fraction, varying from 25–100% during the measurement with an instrument zero recorded at ≥50%; (**b**) highly variable source where methane volume fraction varies by ±25% (i.e., a range of 25–75% about a source initially at 50% volume fraction); (**c**) source with initially unknown and otherwise invariant methane volume fraction.

The repeatability and reduced magnitude of the volume fraction-dependent velocity offset in the DET configuration enables derivation of a simple bias correction function, as plotted in Figure 6. Thus, in a field application, the instrument would simply need to have its velocity zeroed at whatever volume fraction of gas was available in the system (readily accomplished with isolation valves installed on either end of the measurement head); then, the bias correction function could be applied to quantify methane flow rates over a range of volume fractions and velocities. Figure 8 demonstrates achievable methane emission rate uncertainties (i.e., prediction intervals at 95% confidence) over a range of volume fractions and flow rates in three different field application scenarios, depending on the anticipated variability in methane volume fraction of the source. The test data for this figure were collected over three separate days, and during each day methane volume fractions of 25, 50, 75, and 100% were measured at flow velocities ranging from 0 to 2.2 m/s, equating to a methane mass flow rate of up to 11.2 kg/h. 

The first scenario (Figure 8a) represents a case where both the velocity and methane volume fraction of the emitting source may vary significantly during a measurement, spanning the full range of test volume fractions from 25–100%. While this scenario is likely extreme, it could be possible from a storage tank at a site with negligible or intermittent oil production. At such a site, the methane emitted from the tank vent would be diluted by ambient air which entered the tank’s vapor space as the latter contracted due to diurnal temperature cycles. The system could therefore be zeroed once a day at whatever volume fraction was available at that time in the measurement cell. The plotted data then show all possibilities in which the system is zeroed at a volume fraction of ≥50%, and subsequent measurements are made over the full range of conditions after applying the volume fraction-dependent bias function from Figure 6. For this most challenging scenario, the 95% prediction bands indicate a total expected measurement uncertainty of ±2.55 kg/h. 

The second scenario (Figure 8b) assumes that the methane volume fraction of the source of interest will vary by no more than 25% absolute (i.e., a source with 50% methane fraction could range from 25–75%) over the course of the measurement period. The authors’ field measurement experience suggests this as the most common scenario for methane venting from oil production tanks. As in the first scenario, the system could be zeroed at any available volume fraction ≥50%; however, in subsequent measurements the volume fraction is expected to remain within ±25%. In effect, this means that the velocity offset correction of Figure 6 is applied over a narrower range, such that the prediction interval is reduced to ±1.65 kg/h. 

The final scenario (Figure 8c) considers a source with an initially unknown and otherwise invariant methane volume fraction. This would be expected when measuring sources such as well-head or casing vent emissions, where the composition of the vented gas is driven by the geochemistry of the underground formation and not by on-site equipment. In this case, the zero-velocity offset measurement would be made at whatever volume fraction was measured (within the full range of 25–100%), such that the empirical fit from Figure 6 is no longer required. This further reduces the achievable uncertainty to ±0.85 kg/h over the full range of volume fractions and velocities.

## 4. Conclusions

Two different configurations of an intrinsically safe optical measurement system to quantify methane flow rates in hazardous locations at upstream oil production sites were designed, assembled, and characterized. Both the fiber coupled (FC) and detector plus isolation barrier (DET) systems were able to measure the methane volume fraction over a range of 0–100% with uncertainties below 10,000 ppm and velocities from 0–4 m/s at 100% methane fraction with high precision. However, experiments revealed that the Doppler shift-based velocity measurements were subject to a volume fraction-dependent bias error that has not been discussed in previous studies. In the FC configuration, this volume fraction-dependent velocity offset was attributed to modal noise in the return fibers and was found to be unstable when the fibers were manipulated. However, for the DET configuration, the bias function was stable throughout disassembly and reassembly, as well as smaller in magnitude, such that it was possible to fit a simple offset correction that permitted accurate methane flow rate measurement over a range of volume fractions and velocities. Controlled tests considering a range of field deployment scenarios demonstrated the ability of the proposed DET system to quantify methane flow rates within ±0.85 kg/h to ±2.1 kg/h at 95% confidence depending on the assumed variability in the methane fraction of the source. This precision is sufficient for measuring the anticipated vent rates of a range of oil and gas sector vent sources observed in previous field studies, and could be further improved by decreasing the cross-sectional area of the measurement cell, thereby increasing the velocity within it. Finally, it is anticipated that further performance improvements over this initial design should be possible by optimizing the components in the measurement cell to minimize background signals and increase alignment stability for easier industrial deployment.

## Figures and Tables

**Table 1:** Comparison of WMS sensors for subsonic gaseous flows, including the current work.

Paper	Application	Gas	Absorption Range	Flow Range [m/s]	Best Performance ^1^
Lyle et al. 2007a [17]	Wind Tunnel	O_2_	Constant, 2.8%	0–23	±0.25 m/s, SD = 0.22 m/s
Lyle et al. 2007b [18]	Turbine Inlet	O_2_	Constant, 3.5%	25–175	SD = 5.4 m/s
Chang 2010 [19]	Wind Tunnel	H_2_O	Constant, 89%	2.5–18	±0.5 m/s
Kurtz et al. 2016 [20]	Flight Speed Sensor	O_2_	1–2%	5–380	~±26 m/s
Current Work	Methane Venting	CH_4_	0–45%	0–4	±0.16 m/s, SD = 0.07 m/s

^1^ Based on provided information. SD is standard deviation. All performance metrics, including for the current work, are at constant absorption fractions and are thus the best performance to be expected.
